# Differentiation of Human iPS Cells Into Sensory Neurons Exhibits Developmental Stage-Specific Cryopreservation Challenges

**DOI:** 10.3389/fcell.2021.796960

**Published:** 2021-12-14

**Authors:** Rui Li, Patrick Walsh, Vincent Truong, Ashley Petersen, James R. Dutton, Allison Hubel

**Affiliations:** ^1^ Department of Biomedical Engineering, University of Minnesota, Minneapolis, MN, United States; ^2^ Anatomic Incorporated, Minneapolis, MN, United States; ^3^ Division of Biostatistics, University of Minnesota, Minneapolis, MN, United States; ^4^ Department of Genetics, Cell Biology and Development, University of Minnesota, Minneapolis, MN, United States; ^5^ Stem Cell Institute, University of Minnesota, Minneapolis, MN, United States; ^6^ Department of Mechanical Engineering, University of Minnesota, Minneapolis, MN, United States

**Keywords:** cryobiology, induced pluripotent stem cell, differentiation, sensory neurons, controlled rate freezing, cryoprotective agents, Raman spectroscopy

## Abstract

Differentiation of human induced pluripotent stem cells (hiPSCs) generates cell phenotypes valuable for cell therapy and personalized medicine. Successful translation of these hiPSC-derived therapeutic products will rely upon effective cryopreservation at multiple stages of the manufacturing cycle. From the perspective of cryobiology, we attempted to understand how the challenge of cryopreservation evolves between cell phenotypes along an hiPSC-to-sensory neuron differentiation trajectory. Cells were cultivated at three different stages to represent intermediate, differentiated, and matured cell products. All cell stages remained ≥90% viable in a dimethyl sulfoxide (DMSO)-free formulation but suffered ≥50% loss in DMSO before freezing. Raman spectroscopy revealed higher sensitivity to undercooling in hiPSC-derived neuronal cells with lower membrane fluidity and higher sensitivity to suboptimal cooling rates in stem cell developmental stages with larger cell bodies. Highly viable and functional sensory neurons were obtained following DMSO-free cryopreservation. Our study also demonstrated that dissociating adherent cultures plays an important role in the ability of cells to survive and function after cryopreservation.

## Introduction

Human induced pluripotent stem cells (hiPSCs) can be manufactured from a range of somatic cell types and further differentiated into cells valuable for drug discovery (Ko and Gelb, 2014), cell therapy (Yamanaka, 2020), and tissue engineering (Mazzola and Di Pasquale, 2020) applications. However, the generation of hiPSC-derived cells involves complex, protracted, and expensive differentiation protocols that progress through increasingly mature cell stages. There is significant added value in derisking the manufacturing supply chain by being able to efficiently cryopreserve cells at multiple stages along the differentiation trajectory, from the originally isolated somatic tissue through the pluripotent stem cell stage, intermediate progenitor, and differentiated phenotypes, to the terminal mature product. Effective cryopreservation of cells at different developmental stages may not only simplify the multistage production of hiPSC-based technologies but also enable off-the-shelf use of the end product.

Researchers in the field of cryobiology have extensively studied the cell and tissue types at the front end of stem cell-based manufacturing process, from starting materials, such as peripheral blood mononuclear cells (PBMCs) (Nazarpour et al., 2012; Germann et al., 2013; Pi et al., 2020) and fibroblasts (Ragoonanan et al., 2010; Naaldijk et al., 2016) to hiPSCs (Beier et al., 2013; Li et al., 2020) and mesenchymal stromal cells (MSCs) (Hunt, 2011; Pollock et al., 2017). While results of cryopreservation have been reported in some studies of hiPSC-derived cell types (Linville et al., 2020; van den Brink et al., 2020), we lack understanding of scientific principles on formulating cryopreservation media, freezing, thawing, cryoinjury mechanisms for many intermediate and mature cell product phenotypes, whether and how they change from one cell type to another, especially in relation to the expanding variety of cell types in this dynamic field of hiPSC-based manufacturing.

It has been commonly hypothesized among scientists that the more differentiated or more mature the cells are, the more difficult they are to freeze. Cryopreservation practice in the stem cell biology field mirrors this hypothesis, favoring cryopreservation of less differentiated and less mature cell phenotypes along a given differentiation trajectory. Literature describing the cryopreservation of hiPSC-derived cells is predominated by the freezing of intermediate, progenitor-like cell types (McNeish et al., 2010; Nishiyama et al., 2016). The vast majority of academic and industrial use-case literature, in drug screening, for example, completely foregoes cell cryopreservation in favor of continuous cell differentiation and maturation (Egawa et al., 2012; Höing et al., 2012; Burkhardt et al., 2013; Ryan et al., 2013; Yang et al., 2013; Brownjohn et al., 2017; Kondo et al., 2017; Engle et al., 2018; Fujimori et al., 2018). The limited use of cryopreservation is indicative of a knowledge gap that the cutting edge of cryobiological research can begin to address.

In order to effectively cryopreserve cells derived from hiPSCs, it will be important to understand the mechanisms of cell loss when cryopreserving cells along the differentiation and maturation process. As the biological properties of a cell changes as it proceeds along a differentiation trajectory, it will be important to understand how much the cryobiology of that cell changes and whether a given cryopreservation method developed for 1 cell developmental stage can be used for another. These scientific issues will impact the future of hiPSC-based clinical translations.

**FIGURE 1:**
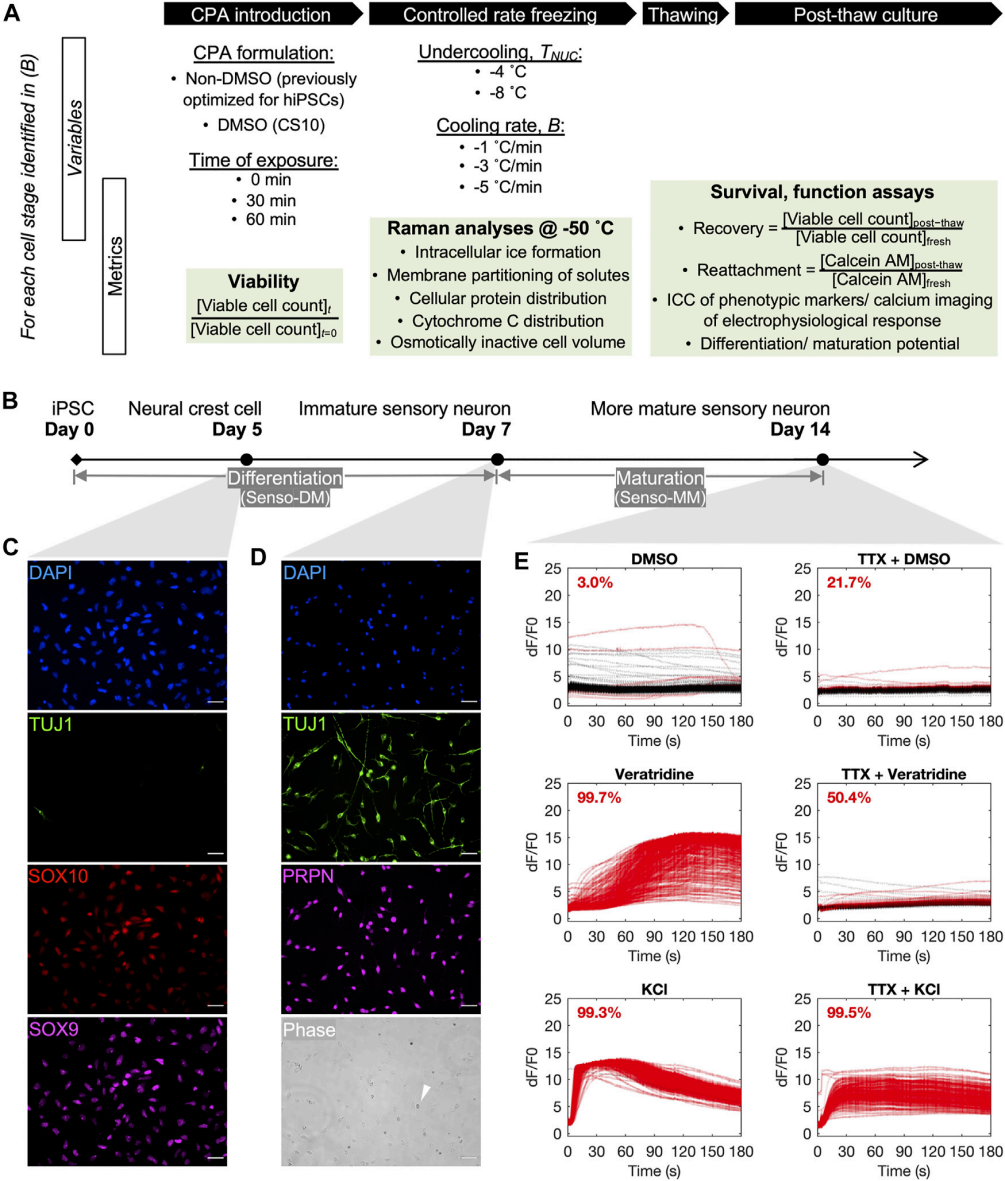
Overview of the cryobiological investigation along a human induced pluripotent stem cell (hiPSC)-to-sensory neuron trajectory. **(A)** Outline of variables and metrics for the analysis, per neuronal cell stage, of cryoprotective agent (CPA) cytotoxicity before freezing, cell response to variation in nucleation temperature (*T*_*NUC*_) and cooling ratio **(B)** during freezing, and cell survival and function after thawing. **(B)** A 14-day differentiation and maturation timeline for cultivating the three developmental stages of neuronal cells of interest for this investigation. **(C)** Immunocytochemistry of day-5 neural crest cells (D5 crest) replated at 100,000 cells/cm^2^, 98.4% TUJ1− SOX9 + SOX10 +. Scale bar: 50 µm. **(D)** Phase contrast and immunocytochemistry of day-7 sensory neurons (D7 SN) replated at 50,000 cells/cm^2^, 98.8% TUJ1 + PRPN + excluding dead cells by morphology (example indicated by white arrow in phase). Scale bar: 50 µm. **(E)** Calcium imaging of day-14 sensory neurons (D14 mSN), seeded at 50,000 cells/cm^2^ on day 7, showing minimal response to dimethyl sulfoxide (DMSO), strong and tetrodotoxin (TTX)-sensitive response to veratridine, and strong response to potassium chloride (KCl). Lines are color-coded red for responder cells and black for nonresponder cells. Proportion of responder cell population indicated per graph.

We present the first systematic evaluation, to our knowledge, of the freezing of discrete differentiation and maturation stages along a cell developmental trajectory, from undifferentiated hiPSC through neural crest cells, immature neurons, to electrophysiologically active sensory neurons, and the influence of stem cell development on the molecular and cellular parameters attributed to cryopreservation success (Figure 1A). In this work, the process parameters are defined as cooling rate and ice nucleation temperature. Particularly, different ice nucleation temperatures represent different degrees of undercooling—reaching temperatures below the intrinsic freezing point of the system—a major cause of cryoinjury in cells (John Morris and Acton, 2013). A dimethyl sulfoxide (DMSO)-free cryoprotective agent (CPA) formulation previously optimized for hiPSC cryopreservation and a DMSO-based formulation that is commercially available are compared. Low-temperature Raman spectroscopy is used during freezing to quantify the response of cells at the different developmental stages. An array of standard (e.g., intracellular ice formation) and novel (e.g., membrane partitioning of CPA solutes) Raman metrics are rendered from the label-free, high-resolution hyperspectral images of live cells. A progression of cell-based assays with increasing functional relevance are used after controlled rate freezing and thawing to characterize the post-thaw outcome for cells cryopreserved at the different stages of differentiation and maturation for a given variation of cooling rate and undercooling.

## Materials and methods

### Induced Pluripotent Stem Cell Culture

The hiPSC line UMN PCBC16iPS (Ye et al., 2013) was maintained using previously published methods between passages 80 and 85. In brief, the cells were cultured as adherent colonies on recombinant human vitronectin (Peprotech) in TeSR-E8 medium (STEMCELL Technologies) and passaged every 4 days as multicellular aggregates using Versene (ThermoFisher Scientific) at a split ratio of 1:16. Cultures were routinely tested for mycoplasma using the MycoAlert PLUS detection kit (Lonza, LT07-701) and karyotyped using G-banding at a 400-band resolution.

### Neuronal Differentiation and Maturation

hiPSCs were differentiated into sensory neurons using a commercially available kit (Senso-DM, Anatomic Incorporated 7007) and further matured (Senso-MM, Anatomic Incorporated 7008) per the instructions of the manufacturer. Briefly, hiPSCs were passaged as 3- to 10-cell aggregates using Versene at a split ratio of 1:20 onto Matrix 1. Cultures were exposed to the first of a series of Senso-DM formulations 24 h later, with subsequent formulations applied daily for 1 week in total. The differentiated cells were dissociated and replated onto Matrix 3 and allowed to mature in Senso-MM media for another 7 days with half media exchange every 2 days. Three different stages of neuronal cells were selected for this investigation on the cryobiological change along this hiPSC-derived lineage (Figure 1B). They were, respectively, the intermediate SOX9-, SOX10-positive, TUJ1-negative neural crest cells obtained on day 5 of this protocol (also abbreviated as D5 crest in later text, Figure 1C), the fully differentiated but immature TUJ1-, PRPH-positive sensory neurons obtained on day 7 (D7 SN, Figure 1D), and the electrophysiologically active and more mature sensory neurons on day 14 (D14 mSN, Figure 1E). This defined 14-day differentiation and maturation process is referred to simply as the (sensory neuron) differentiation trajectory in later text.

### Immunocytochemistry

Cultures were fixed in 3.7% paraformaldehyde (Fisher Scientific) for 10 min, permeabilized in 0.2% Triton X-100 (MilliporeSigma, X100–100 ml) for 10 min, and incubated overnight at 4°C with primary antibodies diluted in a blocking buffer containing 1% bovine serum albumin (Prometheus Protein Biology Products, 25–529) and 0.1% Tween-20 (MilliporeSigma, P7949-100 ml). The cultures were subsequently washed twice with the blocking buffer and incubated for 2 h with secondary antibodies. DAPI (1:1,000, ThermoFisher Scientific) was added for 30 min before washing twice in DPBS. Antibodies used include: SOX9 (1:1,000, MilliporeSigma, AB5535), SOX10 (1:100, R&D Systems, AF2864), TUJ1 (1:500, MilliporeSigma, MAB1637), PRPH (1:1000, Novus Biologicals, NB300137), Alexa Fluor 488 donkey anti-mouse (1:1,000, Invitrogen A-21202), Alexa Fluor 555 donkey anti-goat (1:1,000, Invitrogen, A-21432), Alexa Fluor 647 donkey anti-rabbit (1:1,000, Invitrogen, A-31573). Negative controls included unstained cultures and positively stained cultures known not to express the antigens of interest. Stained cultures were imaged using a Leica DMI6000B microscope and a DFC365FX camera.

### Calcium Imaging

Live D14 mSN cultures were loaded with 2.5 µM Calbryte 520 AM (AAT Bioquest, 20651) and PowerLoad Concentrate (1:100, ThermoFisher Scientific, P10020) for 1 h before imaging. Fluorescent videos of the stained cultures were acquired using a Leica DMI6000B microscope, a DFC365FX camera, and a 10x air objective (NA 0.25, Leica Microsystems) for a duration of 185 s at four frames per second. Varying drug solutions were added to the cultures within the first 10 s of each video. The drugs included veratridine (Tocris, 2918) at a final concentration of 1 µM containing 0.1% DMSO in DPBS, 30 mM potassium chloride (KCl, Millipore Sigma, P5405) containing 0.1% DMSO, 0.1% DMSO as negative control, and 1 µM tetrodotoxin (TTX, Biotium, 00061) intended to block voltage-gated sodium channels.

The image analysis was automated using FIJI. Specifically, individual somata were detected by local maxima-based image segmentation and binary thresholding. Mean intensity was measured over time inside each soma. The intensity profiles were graphed, in terms of *∆F*/*F*_*0*_, where *∆F* is the intensity differential compared with background fluorescence (*F*_*0*_) of the given sample, and the responding cells were quantified using MATLAB. Responders were mathematically defined by the maximum of the first derivative of its fluorescent intensity over time above a constant threshold that was determined by that of the background fluorescence over time.

### Cell Viability in Cryoprotective Agents

A solution of non-DMSO cryoprotective agents (CPAs) was formulated using sucrose, glycerol, L-isoleucine, poloxamer 188 (P188), and human serum albumin as previously optimized for the cryopreservation of hiPSC aggregates (Li et al., 2020). A commercially available DMSO-based CPA solution (CryoStor 10, BioLife Solutions) was used in comparison. Neuronal cells were incubated in the CPA solution at room temperature. Viable and nonviable cell counts were based on membrane integrity using 10 µM acridine orange (AO) and 15 µM propidium iodide (PI) and measured at 0, 30, and 60 min.

### Membrane Fluidity

hiPSC cultures were dissociated with Versene for 8 or 20 min to obtain a suspension of multicellular aggregates or single cells, respectively. Neuronal cell cultures were dissociated with Accumax (Innovative Cell Technologies) for 10, 45, or 90 min at room temperature on day 5, 7, or 14, respectively. Dissociated cells were washed once and stained with a fluorescent pyrene-decanoic acid (PDA) probe (Membrane Fluidity Kit, Abcam, ab189819) at room temperature for exactly 20 min before washing twice and quantification per the instructions of the manufacturer. Phenol red-free DMEM/F12 was used as the suspension media to minimize extrinsic variability between samples. Relative membrane fluidity was measured by the PDA excimer-to-monomer ratio using a Synergy HTX microplate reader with excitation at 360 nm and emission at 400 and 460 nm (BioTek).

### Cell Diameter

Neuronal cells of the different developmental stages were dissociated as described above. Live cells were stained immediately after dissociation using AO and imaged in a hemocytometer (Hausser Scientific) using a Zeiss Axioskop 50 microscope with a 10x air objective (Plan NeoFluar, NA 0.30; Carl Zeiss). The cross-sectional area (*A*) of each cell was quantified using FIJI via thresholding and boundary recognition. Diameter of the fresh cell (*D*_*fresh*_) was estimated using the following equation.

$$D_{fresh}=\;2\sqrt{\frac{A}{\pi }}$$

### Low-Temperature Confocal Raman Spectroscopy

Cell samples were prepared in the form of single-cell suspension and frozen at a controlled rate for analysis by Raman spectroscopy following a previously published method (Li et al., 2020; Yu et al., 2021). Specifically, defined cooling rates of −1, −3, and −5°C/min and ice nucleation temperatures of −4°C and −8°C were used as variables of the freezing profile. Data acquisition was performed at −50°C when sample temperature had stabilized for a minimum of 5 min. Raman spectroscopic measurements were made using WITec Confocal Raman Microscope Alpha 300R with UHTS spectrometer and DV401 CCD detector with 600/mm grating, a 532-nm Nd:YAG laser, and a 100x air objective (NA 0.90, Nikon Instruments).

Raman heat maps of different substances of interest were, respectively, rendered pixel-wise by integrating the Raman spectra under the peak at their characteristic wavenumbers (Supplementary Table S1). An integration time of 0.2 s was used to scan each 333-nm-by-333-nm pixel. The rendered Raman heat maps were spatially deconvolved using a theoretical point spread function of the instrument prior and quantitatively analyzed using WITec Project FOUR and FIJI, where pixels belonging to the different substances were, respectively, identified by spectral bandpass filtering, thresholding, and boundary recognition. Moran’s I calculation of amide I and cytochrome C signals was performed using GeoDa, where spatial dispersion vs. autocorrelation was determined between −1 and 1 using a spatial weight matrix based on eight nearest neighbors. Similar to fresh cells, cell diameter during freezing was quantified based on the cross-sectional area of the cell as delineated by the amide I and cellular hydrogen bond signals. The effect of freezing on the osmotically inactive cell volume was represented by the diameter of frozen cells normalized to the mean diameter of fresh cells for each given cell stage and freezing condition.

### Controlled Rate Freezing and Thawing

Cells of different stages were dissociated as described above and suspended in a collection buffer containing P188 (Li et al., 2020). A twice-concentrated CPA solution previously optimized for hiPSC cryopreservation (Li et al., 2020) was added at equal volume. The mixture was incubated at room temperature for 1 h and subsequently frozen in cryogenic vials (Nunc CryoTubes, ThermoFisher Scientific) using a controlled rate freezer (Kryo 560–16, Planer) following the steps listed below. Similar to Raman experiments, a cooling rate, *B*, of −1°C/min, −3°C/min, or −5°C/min and ice nucleation temperature, *T*_*NUC*_, of −4°C or −8°C were used (see Supplementary Figure S1 for the sample cooling profiles).

1. Starting temperature 20°C.
2. −10°C/min to *T*_*NUC*._
3. Hold at *T*_*NUC*_ for 15 min to equilibrate sample and chamber temperatures.
4. Induce ice nucleation manually by briefly spraying liquid nitrogen onto vials using a Cryogun (Brymill) for accurate control of the nucleation temperature.
5. *B*°C/Min to −60°C.
6. −10°C/Min to −100°C.

A sample temperature inside a replicate “dummy” vial was logged for each experiment using the built-in thermocouple of the controlled rate freezer inserted via a bored vial cap. Frozen vials were stored in the liquid phase of liquid nitrogen for 24 h prior to thawing in a 37°C water bath for 2.5 min. Thawed cells were immediately used for post-thaw quantifications.

### Post-Thaw Quantification of Cell Survival and Function

Post-thaw cell recovery rate was defined as the percent ratio of viable cell count immediately after thawing and viable cell count immediately before CPA addition prefreeze, based on cell membrane integrity distinguished by AO stain and PI exclusion. Post-thaw cell attachment rate was defined as the percent ratio of adhered, metabolically active cell culture in 24 h after thawing and that of 24 h after passaging fresh cells, where cultures were stained with calcein AM (1:1,000, Corning 354217) at 37°C for 30 min before quantified using Synergy HTX microplate reader with 480-nm excitation and 528-nm emission filters. Post-thaw cell function was measured, respectively, by the ability of cryopreserved D5 crest to continue differentiating to TUJ1-positive, PRPH-positive sensory neurons 48 h later, using immunocytochemistry as described above, of cryopreserved D7 SN to produce calcium response in 7 days, using calcium imaging as described above, and of cryopreserved D14 mSN to retain calcium response in 24 h, all in comparison with fresh cells through the corresponding manipulation with only cryopreservation procedures removed.

### Statistics

Independent biological replicates were used with sample size specified in the *Results* section per dataset. Power analysis was performed to ensure sufficient sample size to achieve a power of 0.95. Error bars represent 95% confidence intervals unless otherwise noted. Two-tailed Student’s *t*-tests were performed for two-sample comparisons, unless otherwise noted. ANOVA with Bonferroni correction was performed for comparisons of multiple samples, with the exception of Kruskal–Wallis ANOVA performed for sample populations with non-normal distribution. The null hypothesis was defined as no statistical difference between the parameters (e.g., means) for any pair of groups or between the experimental group and control group. The null hypothesis was rejected, and differences were considered statistically significant for a *p*-value less than 0.05.

## Results

### Non-DMSO Solution Composed of Sucrose, Glycerol, L-Isoleucine, P188, and HSA was Superior to DMSO-Based Commercial Solution in Maintaining Neuronal Cell Viability Before Freezing

**FIGURE 2:**
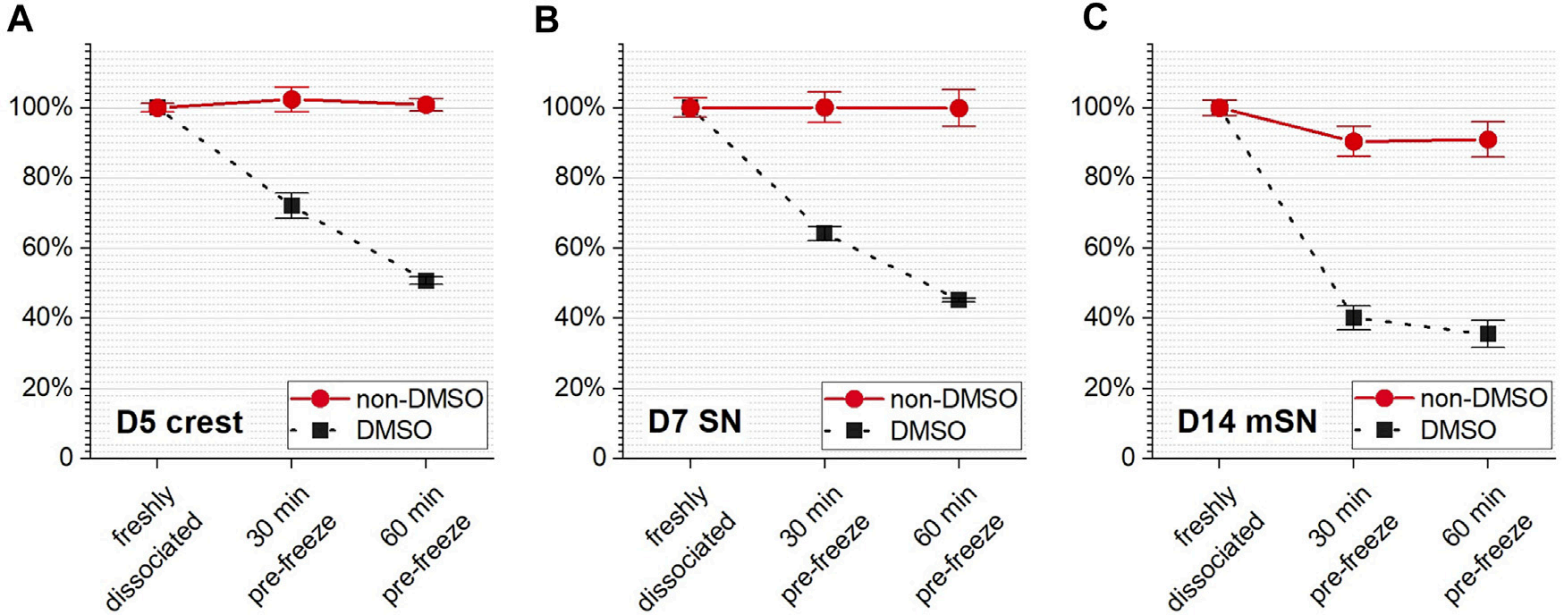
Cell survival rate during CPA incubation prior to cryopreservation as monitored over time of exposure to the non-DMSO CPA solution vs. the DMSO-based solution for **(A)** D5 crest, **(B)** D7 SN, and **(C)** D14 mSN, respectively. Data are represented as mean ± 95% confidence interval. *n* = 4.

Cells are cryopreserved in specialized solutions containing cryoprotective agents (CPAs). These solutions are not physiological, and cell losses can result from exposure to these solutions. Different cell types can have different responses to being exposed to the same CPA formulation, and different CPA formulations can have different levels of cytotoxicity for a given cell type. In this study, a non-DMSO CPA solution composed of sucrose, glycerol, L-isoleucine, P188, and HSA and previously developed for hiPSCs (Li et al., 2020) was compared with a DMSO-based solution commercially available and commonly used for hiPSC-derived cells (i.e., CS10) in terms of their ability to maintain the viability of different stages of neuronal cells before freezing. In order to quantify the toxicity of the cryoprotective solutions, viable cell count was tracked, via membrane exclusion fluorescent assay, for 1 h from the introduction of non-DMSO CPA and DMSO solutions before freezing for D5 crest, D7 SN, and D14 mSN, respectively (Figure 2).

Compared with the non-DMSO CPA, the DMSO solution resulted in significant cell loss by both loss of membrane integrity and lysis (high cell count with compromised membrane and low total cell count, raw data not shown) for all cell stages within 30 min of exposure. After 1 h of prefreeze incubation in DMSO, all cell stages experienced close to (D5 crest, *p* > 0.05) or greater than (D7 SN and D14 mSN, *p* < 0.05) 50% cell loss. Notably, the cell loss increased over time regardless of cell stage. At both the 30- and 60-min timepoints, it was found with statistical significance (*p* < 0.05) that the farther the cell stage in differentiation and maturation, the lower the cell stability in DMSO.

In contrast, no significant change in viable cell count (*p* > 0.05) was seen in either D5 crest or D7 SN throughout the 1-h incubation in the non-DMSO solution. While the cell survival rate of D14 mSN decreased from 100% to approximately 90% within the first 30 min of non-DMSO CPA exposure, their viability remained statistically unchanged (*p* > 0.05) for the next 30 min until freezing. Notably, regardless of cell stage, the non-DMSO CPA formulation was significantly superior (*p* < 0.05) to the DMSO-based formulation in stabilizing these neuronal cells and maintaining their viability in the time period prior to the start of freezing.

### Membrane Fluidity and Cell Size Fluctuations During Neuronal Cell Differentiation and Maturation Inferred Cell Stage-Dependent Sensitivity to Undercooling and Cooling Rate

**FIGURE 3:**
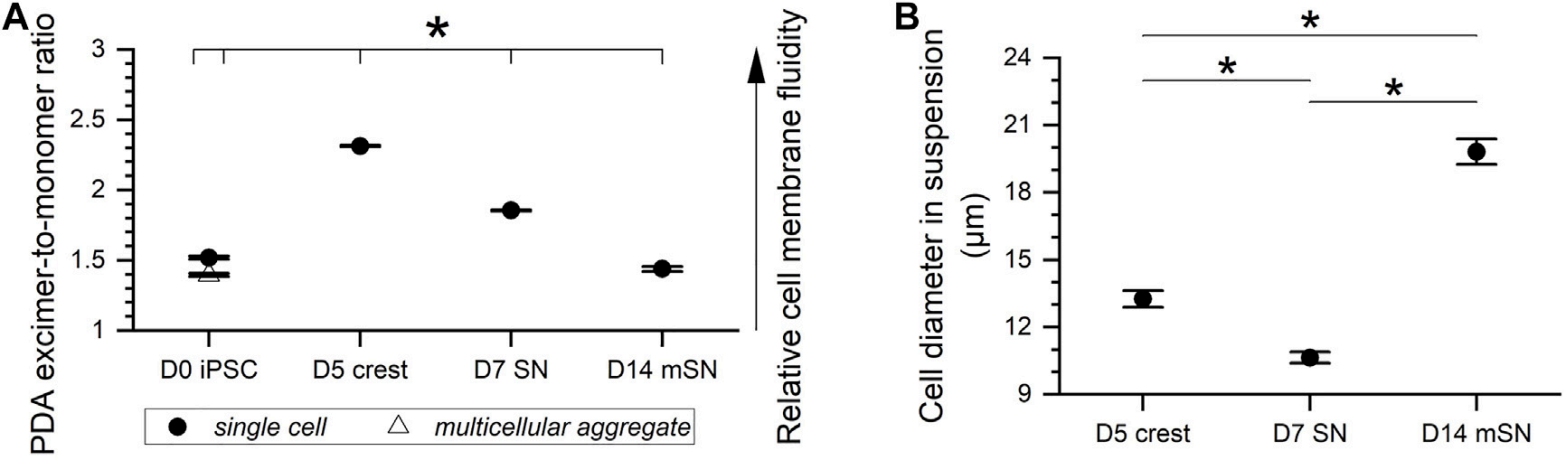
Membrane fluidity and cell size fluctuation along hiPSC derivation to sensory neurons. **(A)** Cell membrane fluidity at different stages of the sensory neuron differentiation and maturation process with all statistically significant pairwise differences. Data are represented as mean ± 95% confidence interval. *n* = 9. *p < 0.05. **(B)** Diameter of D5 crest vs. D7 SN vs. D14 mSN dissociated from culture and suspended in growth medium. Data are represented as mean ± 95% confidence interval. Range of *n*: 60–98. *p < 0.05.

As one possible factor influencing the sensitivity of the cells to freezing (Giraud et al., 2000; Blesbois et al., 2005), membrane fluidity was measured for each cell stage, upon dissociation and in suspension, along the 14-day differentiation trajectory (Figure 3A). These measurements included hiPSCs in the forms of single cells and multicellular aggregates, D5 crest, D7 SN, and D14 mSN in the form of single cells. Based on PDA excimer-to-monomer ratios, membrane fluidity of the cells underwent significant changes as they became more differentiated and mature. Interestingly, within the 5 days of differentiation from hiPSCs to neural crest cells, the relative membrane fluidity increased by more than 50%. In the next 48 h from neural crest cells to fully differentiated sensory neurons, the change in membrane fluidity inverted direction by decreasing approximately 20%. This decrease continued, at an overall slower rate than earlier, for another week as the neurons matured. PDA assay also showed that hiPSC aggregates had significantly lower membrane fluidity than their single cell counterparts, inversely correlated with the significantly greater sensitivity to undercooling that was found by previous studies (Li et al., 2018, 2020). Notably, the fluctuation in membrane fluidity along this differentiation trajectory was not unidirectional, increasing during the initial pluripotent-to-progenitor step but decreasing from progenitor through maturation based on the selected discrete cell stages. This fluctuation in membrane fluidity suggests likely fluctuation in the sensitivity of neuronal cells to undercooling, one category of freezing sensitivity in response to a specific parameter of freezing–nucleation temperature.

In addition to membrane fluidity, cell size was considered another potential intrinsic factor influencing cell sensitivity to freezing, specifically cell sensitivity to another parameter of freezing–cooling rate. Larger cells with smaller surface-to-volume ratio may contain more intracellular water during cooling and, thus, higher potential for intracellular ice (Gao and Critser, 2000; Hubel, 2018). The diameter of the cells in suspension was observed to fluctuate significantly from one cell stage to the next along this differentiation trajectory (Figure 3B). D7 SN was found with the smallest cell diameter in suspension at 10.6 µm (± 0.2 µm, 95% confidence interval), followed by D5 crest at 13.3 µm (± 0.4 µm), and D14 mSN with the largest cell diameter at 19.8 µm (± 0.6 µm). Changes in soma size have been known in literature to occur during neuronal development (Nixdorf-Bergweiler, 1998; Deshpande et al., 2017). Notably, the fluctuation in cell size was not unidirectional from neuronal differentiation to maturation. However, somata (or cell body) enlargement over the course of neuronal maturation observed in this study was also consistently observed in stem cell biology practices. In subsequent experiments, freezing sensitivity (i.e., undercooling and cooling rate sensitivity) of these neuronal cells was observed using low-temperature Raman spectroscopy and post-thaw cell-based assays to vary by the different stages of development, where correlation between membrane fluidity and cell responses to undercooling and between cell size and cell responses to cooling rate variation was found.

### Sensitivity to Undercooling and Mechanisms of Damage Varied by Cell Stage

D5 crest, D7 SN, and D14 mSN were frozen in the non-DMSO or DMSO-based solution under a confocal Raman microscope. The cells were subjected to different degrees of undercooling, where the freezing process was initiated with ice nucleation induced at different temperatures, −4 vs. −8°C, and subsequently cooled at a constant rate of −1°C/min. In each frozen cell, intracellular ice formation, membrane partitioning of solutes, cellular protein distribution, and cytochrome C distribution were quantified (see Supplementary Figure S2 for definition and illustrative examples of different values of the corresponding metrics). Cryobiological analysis of the frozen cells revealed mechanisms of damage, or the absence thereof, at each cell stage in response to the varied CPA solutions and varied undercooling conditions.

**FIGURE 4:**
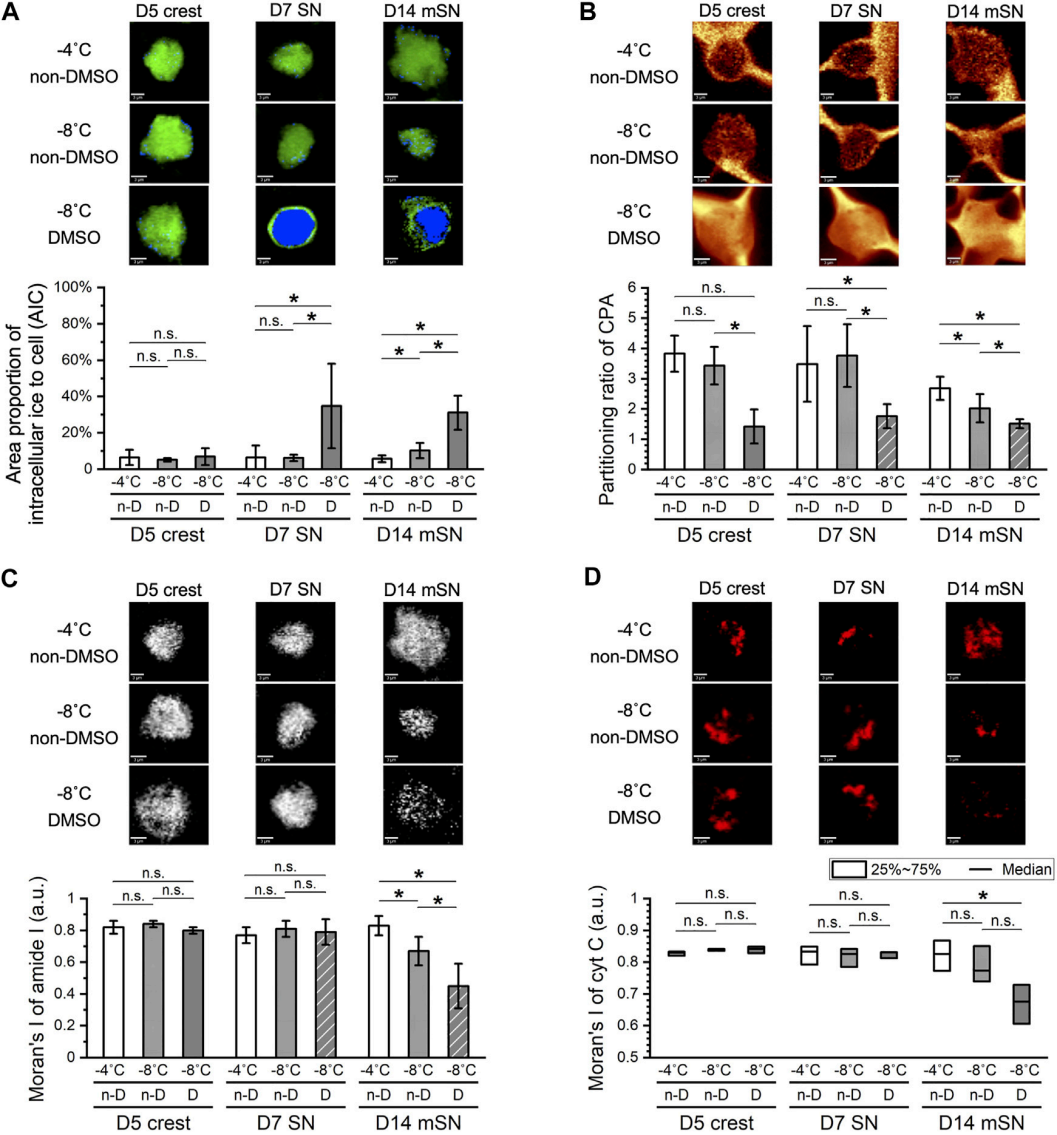
Effects of undercooling on freezing behaviors of D5 crest, D7 SN, and D14 mSN, respectively, as observed by low-temperature Raman spectroscopy, comparing different ice nucleation temperatures between −4°C and −8°C. Data are represented as mean ± 95% confidence interval. *n* = 10, except for striped columns representing measurements of subset population *n =* 5 for D7 SN and *n =* 7 for D14 mSN due to interference of intracellular ice. n-D, non-DMSO; D, DMSO. n.s.: *p* > 0.05; *p < 0.05 (see Supplementary Figure S2 for method illustration of each metric.). **(A)** Intracellular ice formation (blue) significantly increased with greater undercooling only in the case of D7 SN in the DMSO solution and D14 mSN in both non-DMSO and DMSO solutions; no significant change otherwise. **(B)** Membrane partitioning of non-DMSO CPA significantly decreased in D14 mSN subject to greater undercooling, partitioning of DMSO lower than that of non-DMSO CPA across all three cell stages. **(C)** Cellular integrity in terms of spatial autocorrelation of the amide I signal of protein decreased significantly for D14 mSN with greater undercooling and decreased further in the DMSO solution; no significant loss of integrity in D5 crest or D7 SN. **(D)** Significant cytochrome C (cyt C) release was observed for D14 mSN in the DMSO solution but not for any cell stage in the non-DMSO CPA. Kruskal–Wallis ANOVA performed for non-normal distribution of cyt C Moran’s I values.

While extracellular ice crystal formation is an element of slow freezing, the formation of intracellular ice crystals of large quantity or size can be a lethal event. In terms of intracellular ice formation (Figure 4A), D5 crest showed no statistically significant response to the different ice nucleation temperatures or different CPA formulations. When the non-DMSO CPA was used, D7 SN also showed minimal intracellular ice that was consistent between different ice nucleation temperatures. However, a significantly greater amount of intracellular ice crystals was found in the D7 SN frozen in DMSO, where intracellular ice took up nearly the entirety of cytoplasmic space (abbreviated as chunky intracellular ice from here on) for approximately 30% of the cell population. Moving further along the cell developmental trajectory, when D14 mSN was frozen in the non-DMSO solution, it had significantly more intracellular ice crystals with greater undercooling. When D14 mSN was frozen in the DMSO solution, intracellular ice took a different form, chunky rather than dispersed. As intracellular ice formation has been found to be indicative of sensitivity to undercooling in our earlier studies (Li et al., 2018, 2020), these new results from D5 crest, D7 SN, and D14 mSN suggested not only that undercooling sensitivity varied between the different cell stages but also that the sensitivity of a given cell stage was different in the two CPA formulations.

Cell populations found with substantial quantity of intracellular ice (i.e., five of 10 cells for D7 SN frozen in DMSO; three of 10 cells for D14 mSN frozen in DMSO) were omitted from some of the following analyses (Figures 4B,C) due to interference of ice signal and lack of the intracellular Raman signals of interest. As a result, this dataset reveals other mechanisms of damage in cells, while intracellular ice formation was relatively well inhibited. Membrane partitioning of solutes (i.e., non-DMSO CPA or DMSO) describes the steady state of mass transport across the plasma membrane and is mathematically represented as the ratio of the extracellular concentration to the intracellular concentration. While a non-penetrating CPA (e.g., sucrose) is expected to remain out of the cytoplasm, a healthy cell membrane should also be able to partition a penetrating CPA (e.g., glycerol, DMSO), where the intracellular concentration is typically lower than the extracellular concentration of that CPA unless active transport is involved.

In terms of partitioning ratio of the non-DMSO CPA (Figure 4B), D5 crest and D7 SN both showed no significant change upon greater undercooling, whereas D14 mSN exhibited significantly weaker membrane partitioning when subjected to lower ice nucleation temperature. One possible explanation for the decrease in partitioning is impaired barrier function of the plasma membrane of the D14 mSN upon undercooling, corresponding to the higher intracellular ice formation of the cells, more punctate protein distribution, and cytochrome C release. Comparing non-DMSO CPA and DMSO, membrane partitioning of DMSO was found to be significantly (up to 2.4-fold) weaker than that of the non-DMSO CPA for the neuronal cell stages investigated. While it cannot indicate or rule out the possibility of membrane impairment by DMSO, the consistent trend across different cell stages clearly showed that cell membranes have a lower barrier effect to DMSO than glycerol and other non-DMSO CPA molecules. As extracellular ice forms and grows with cooling, cells dehydrate, and intracellular solutes become more concentrated. When DMSO was used, the neuronal cells were more likely subjected to stresses of high intracellular CPA concentration than when non-DMSO CPA was used.

Moran’s I of amide I signal in cells was used to quantify the distribution of cellular proteins. As shown in Figure 4C, D14 mSN was the only cell stage among those tested to be affected by the stresses of undercooling and DMSO exposure on a protein level. When chunky intracellular ice was successfully inhibited, D5 crest and D7 SN exhibited consistently high, uniform distribution of proteins during freezing regardless of ice nucleation temperature or CPA formulation, whereas D14 mSN showed more punctate distribution of proteins upon greater undercooling, a sign of cellular disintegration, and became further disintegrated when frozen in the DMSO-based solution. Moran’s I of cytochrome C signal in cells measured the potential of post-thaw mitochondrial apoptosis and necrosis. As shown in Figure 4D, D14 mSN was the only cell stage showing cytochrome C release when frozen in DMSO, although the cytochrome C distribution of D14 mSN did not respond significantly to undercooling.

Summarizing these four cryobiological metrics, intracellular ice was the only observed mechanism of freezing damage in the earlier cell stages (i.e., D5 crest, D7 SN), where chunky intracellular ice was particularly prominent in D7 SN frozen in the DMSO solution. Further downstream of this differentiation trajectory, higher undercooling sensitivity manifested in the more mature cell stage (i.e., D14 mSN). For D14 mSN frozen in the non-DMSO CPA, significantly higher quantity of small intracellular ice was found and likely resulted in impaired membrane barrier function, higher intracellular CPA concentration, and consequently partially disintegrated cellular structure. For D14 mSN frozen in DMSO, besides the damage of chunky intracellular ice, they suffered from cellular disintegration and cytochrome C release. Similar to previously reported observations in hiPSCs (Li et al., 2018, 2020), compared with the DMSO-based formulation, the non-DMSO CPA formulation utilized here reduced damage in cells that were subjected to lower ice nucleation temperature, extrinsically mitigating the cells’ sensitivity to undercooling. The overall increase in cell sensitivity to undercooling from a multitude of mechanisms along this sensory neuron differentiation trajectory correlated with the decrease in cell membrane fluidity from D5 crest through D14 mSN (Figure 3A), confirming membrane fluidity as a likely factor contributing to the intrinsic sensitivity of the cells to undercooling.

### Sensitivity to Cooling Rate Varied Among Cell Stages and Correlated with Cell Size

**FIGURE 5:**
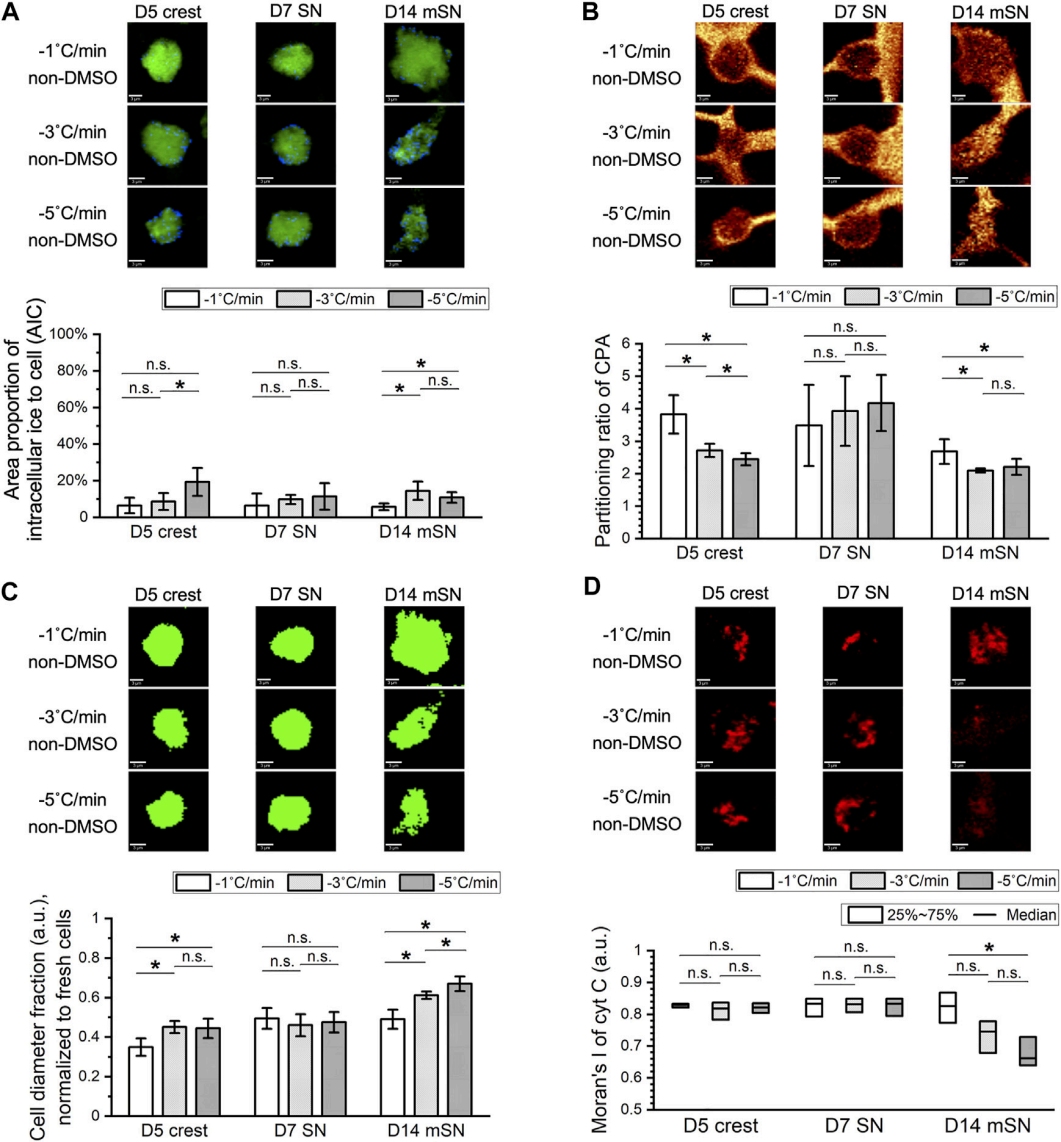
Effects of cooling rate on freezing behaviors of D5 crest, D7 SN, and D14 mSN, as observed by low-temperature Raman spectroscopy, comparing different cooling rates between −1°C/min, −3°C/min, and −5°C/min, respectively. Data are represented as mean ± 95% confidence interval. *n* = 10. n.s.: *p* > 0.05; *p < 0.05 (see Supplementary Figure S2 for method illustration of each metric.) **(A)** Intracellular ice formation (blue) increased with the faster cooling rates in D5 crest and D14 mSN; no significant difference across cooling rates in D7 SN. **(B)** Membrane partitioning of non-DMSO CPA significantly decreased in D5 crest and D14 mSN with the faster cooling rates; no significant difference across cooling rates in D7 SN. **(C)** Loss of cell volume as a result of freezing intensified with the faster cooling rates for D5 crest and D14 mSN; no significant difference across cooling rates in D7 SN. **(D)** Significant cytochrome C (cyt C) release was observed for D14 mSN with the faster cooling rates but not for D5 crest or D7 SN at any of the cooling rates. Kruskal–Wallis ANOVA performed for non-normal distribution of cyt C Moran’s I values.

Parallel to the examination of the effect of undercooling, the effect of different cooling rates on cell behaviors during freezing was also examined. D5 crest, D7 SN, and D14 mSN were subjected to cooling rate that varied between −1°C/min, −3°C/min, and −5°C/min, and their potential mechanisms of damage, or the absence thereof, were analyzed using low-temperature Raman spectroscopy (Figure 5). The non-DMSO CPA formulation previously optimized for hiPSCs was used throughout these experiments. Interestingly, D5 crest and D14 mSN both experienced freezing damages that varied by cooling rate, whereas D7 SN consistently exhibited no statistically significant change across the selection of Raman-based cryobiological analyses regardless of the cooling rate.

In terms of intracellular ice formation (Figure 5A), with ice nucleation temperature held constant at −4°C, only small rather than chunky intracellular ice was observed. D5 crest maintained minimal levels of intracellular ice with the slower cooling rates (i.e., −1°C/min and −3°C/min) and contained a significantly greater quantity of ice with the fastest cooling rate tested (i.e., −5°C/min). D14 mSN maintained a minimal level of intracellular ice with the slowest cooling rate (i.e., −1°C/min) but had a significant increase in intracellular ice with the faster cooling rates starting at −3°C/min. In terms of membrane partitioning of the non-DMSO CPA (Figure 5B), the faster cooling rates (i.e., −3°C/min and −5°C/min) resulted in significantly weaker partitioning in both D5 crest and D14 mSN than the slower cooling rate (i.e., −1°C/min). The weaker partitioning subjected the cells to higher concentrations of intracellular CPA and possibly indicated impairment of the membrane’s barrier function.

As extracellular ice grows during cooling, cells are expected to dehydrate and reduce in volume and diameter. As greater dehydration is commonly expected with slower cooling rates, where water takes longer to leave the cell via osmosis and balance the concentration gradient of solutes across the plasma membrane, the reduction of cell diameter as a result of dehydration is expected to be less upon faster cooling rate for a given cell type. Interestingly, when cell diameter was measured during freezing by the distribution of Raman cellular hydrogen bond and amide I signals and normalized to the diameter of the cells before freezing, this inverse correlation between cell diameter fraction and cooling rate was not found for any of the neuronal cells of interest (Figure 5C). For D7 SN, no significant difference in cell diameter fraction was observed in response to the cooling rate variations. For D5 crest and D14 mSN, the faster cooling rates (i.e., −3°C/min and −5°C/min) resulted in significantly greater loss of cell diameter than the slower cooling rate (i.e., −1°C/min). Comparing the ratio in spectral intensity of the C–H stretching peak to that of the broad O–H stretching band between the cells frozen at different cooling rates, no distinct difference in the hydration level of the cells was observed (data not shown). Therefore, the correlation between intensified loss of cell diameter and faster cooling rate was likely *not* a direct contradiction to the common expectation described above but suggesting another mechanism of cell damage at play. Combined with more intracellular ice, weaker membrane partitioning, and cytochrome C release observed upon faster cooling, greater loss of cell diameter in the frozen D5 crest and D14 mSN likely indicated loss of cell contents and either necrosis or apoptosis of the cells.

In addition, cytochrome C release was observed in D14 mSN at the faster cooling rates but not in D5 crest (Figure 5D). Summarizing the number of mechanisms of freezing damages observed per cell stage and the extent of its response to cooling rate variation, D14 mSN was found to be the most susceptible to fast cooling rates, followed by D5 crest, followed by D7 SN. This trend was consistent with the original cell sizes measured upon dissociation from fresh culture and suspension in the cell growth media (Figure 3B). The larger cells (i.e., D14 mSN at ∼20 µm in diameter > D5 crest at ∼13 µm in diameter) with lower surface-to-volume ratio required slower cooling rate (i.e., −1°C/min) to prevent damages from both intracellular ice formation and dynamic osmotic stresses, whereas the smaller cell (i.e., D7 SN at ∼11 µm in diameter) minimized intracellular ice formation, maintained membrane integrity, and prevented disintegration of cellular proteins across the range of cooling rates investigated (i.e., from −1 to −5°C/min). While this correlation between cell size and cooling rate sensitivity, in general, was consistent with what has been demonstrated in literature, the absence of chunky intracellular ice and the analysis of membrane partitioning and cell volumetric loss in the present investigation presented an alternative mechanism of damage in large cells during fast cooling, in contrast with and a supplement to the commonly hypothesized, generalized mechanism that has been based solely on the formation of optically visible (i.e., chunky) intracellular ice crystals.

### Successful Cryopreservation Relied on Good Cryoprotection and Compatible Process

The next phase of this investigation involved evaluating the performance of these cryopreserved cells upon vial-based controlled rate freezing in the non-DMSO CPA formulation, with ice nucleation varied between −4°C and −8°C and cooling rate varied between −1°C/min, −3°C/min, and −5°C/min. Post-thaw cell recovery was measured by membrane exclusion fluorescent assay to assess membrane integrity. Post-thaw cell reattachment was quantified by measuring the esterase activity of adherent cells. Post-thaw differentiation to sensory neurons was tested for cryopreserved D5 crest to assess their differentiation potential; post-thaw maturation to electrophysiologically active neurons was examined for cryopreserved D7 SN to assess the ability to reach a more mature state; post-thaw electrophysiological response was intended for cryopreserved D14 mSN to assess the ability to retain the level of maturity. The different forms of post-thaw assay progressed in the aforementioned order toward representing the ability of a given cryopreservation method to fulfill the intended utility of the cells.

**FIGURE 6:**
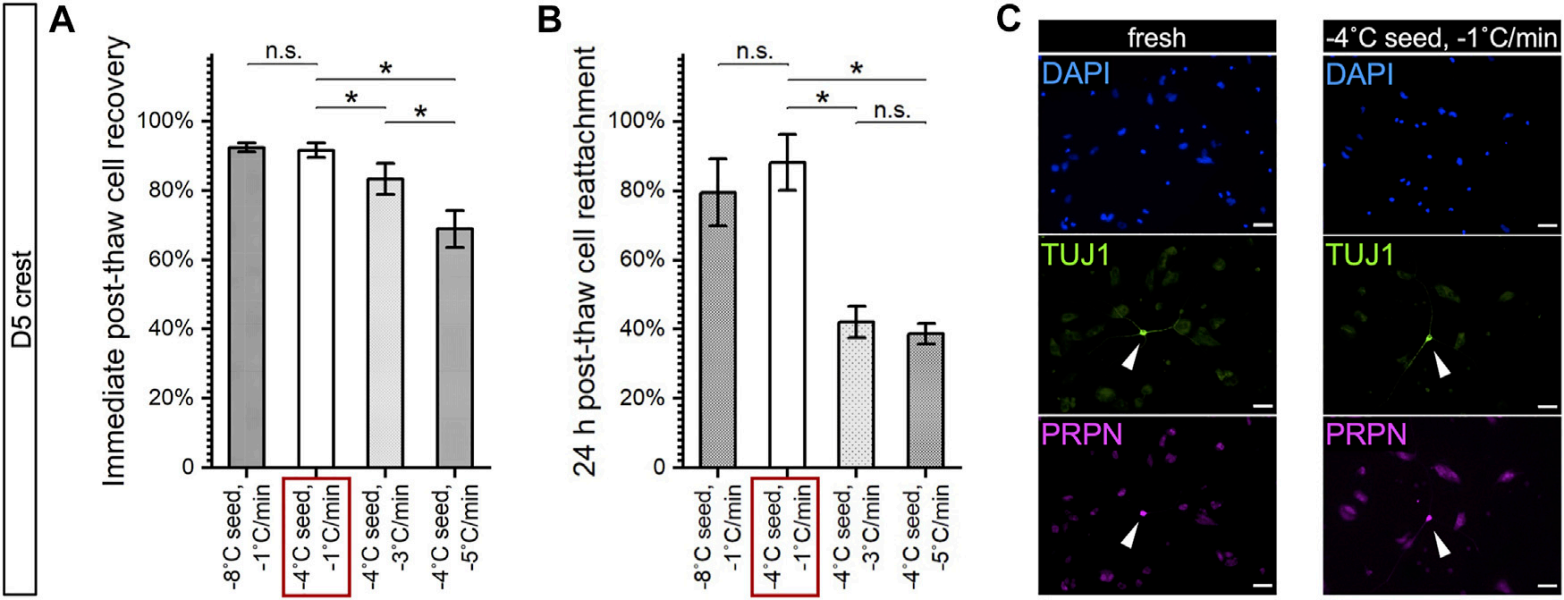
Post-thaw survival and function of cryopreserved D5 crest under varying nucleation temperatures and cooling rates. *p < 0.05; n.s.: *p* > 0.05. Red box: best-case parameters tested with the highest recovery and reattachment. **(A)** Post-thaw recovery of D5 crest showing no significant change with greater undercooling but trending significantly lower with faster cooling rate. *n* = 4. **(B)** Post-thaw reattachment of D5 crest showing similar trends as recovery but more distinct cell damage with cooling rates of −3°C/min and −5°C/min. *n* = 4. **(C)** Immunocytochemistry 48 h after re-culture of dissociated (fresh) or cryopreserved (best-case) D5 crest showing <1% TUJ1+, PRPN+ (white arrowheads) neuron differentiation, and a vast majority of resulted cell population with crest-like morphology and low-level, cytoplasmic TUJ1 and PRPN staining. Scale bar: 100 µm.

For D5 crest, both post-thaw recovery (Figure 6A) and post-thaw reattachment (Figure 6B) demonstrated a lack of cell sensitivity to undercooling and significant cell loss to faster cooling rates. This trend was consistent with the Raman results described earlier, indicating that a cooling rate of −1°C/min or slower was required for D5 crest to prevent loss in membrane integrity or cell adhesion. Despite the successful cryoprotection demonstrated in the combination of −1°C/min cooling and −4°C ice nucleation by post-thaw recovery greater than 90% and post-thaw reattachment of 88%, the cryopreserved D5 crest failed to produce sensory neurons after subsequent days of neuronal induction (Figure 6C). However, notably, the fresh control group of D5 crest that was dissociated and replated into culture also lost its ability to differentiate into TUJ1-positive, PRPH-positive neurons. Dissociating the cells on day 5 interrupted the adherent culture and deviated from the originally continuous 7-day differentiation process, which proved to be detrimental to cryopreserving the D5 crest despite the otherwise effective freezing protocol and CPA formulation.

**FIGURE 7:**
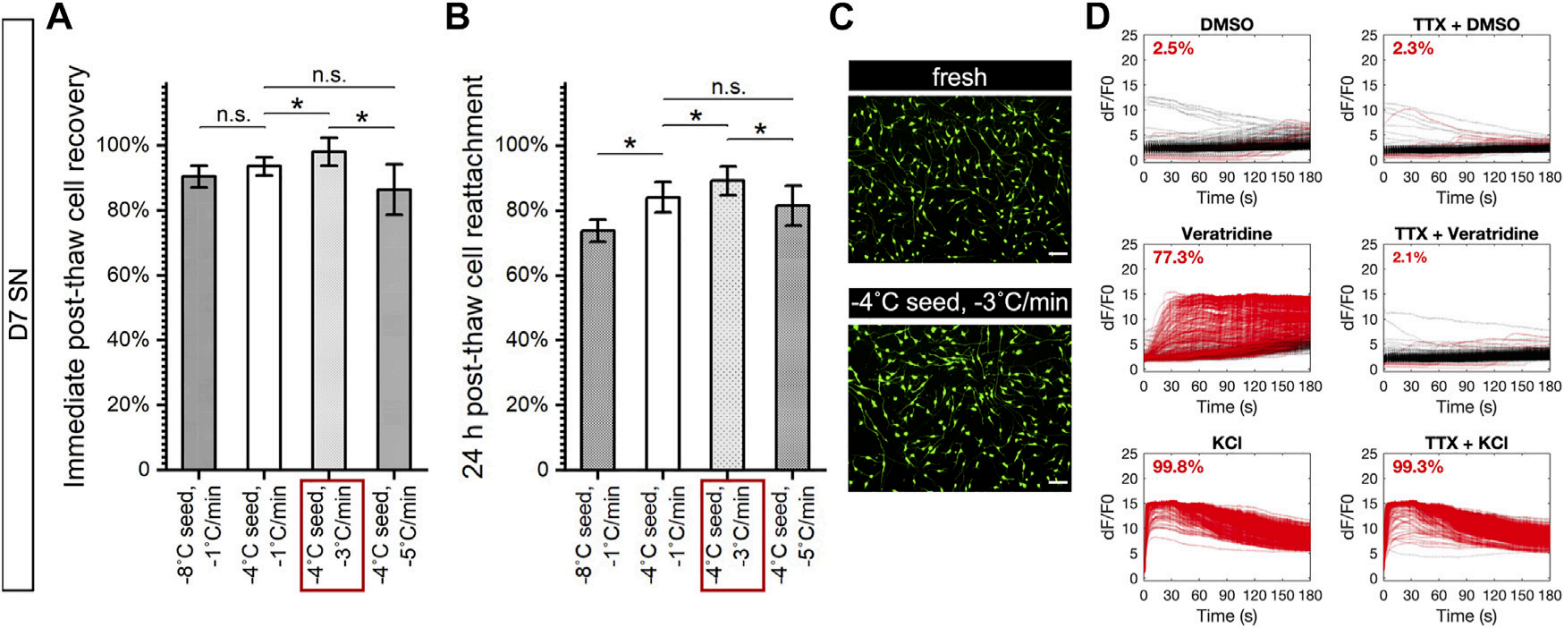
Post-thaw survival and function of cryopreserved D7 SN under varying nucleation temperatures and cooling rates. Data are represented as mean ± 95% confidence interval. *p < 0.05; n.s.: *p* > 0.05. Red box: best-case parameters tested with the highest recovery and reattachment. **(A)** Post-thaw recovery of D7 SN showing no significant change with greater undercooling and the best cooling rate at −3°C/min with statistical significance. *n* = 4. **(B)** Post-thaw reattachment of D7 SN showing similar trends as recovery but more distinct cell damage with lower nucleation temperature (−8°C). *n* = 4. **(C)** Calcein AM-stained culture 24 h after replating dissociated (fresh) or cryopreserved (best-case) D7 SN showing high confluence and normal morphology of immature neurons. Scale bar: 100 µm. **(D)** Calcium imaging of post-thaw culture after 7-day maturation of D7 SN cryopreserved with −4°C nucleation and −3°C/min cooling rate, showing little to no response to 0.1% DMSO (negative control), positive response to 1 µM veratridine that was inhibited by TTX, and positive response to 30 mM KCl that was unaffected by TTX. Red line: responder cell; black line: nonresponder cell. Proportion of responder cell population indicated per graph. Range of *n* = 417–662.

For D7 SN, post-thaw recovery (Figure 7A) demonstrated a lack of cell sensitivity to undercooling that was consistent with the Raman results described earlier. However, post-thaw recovery showed a statistically significant difference between different cooling rates that was not revealed by the Raman measurements. In addition, post-thaw reattachment of D7 SN (Figure 7B) demonstrated not only cell sensitivity to cooling rate but also to undercooling that was not reflected by the Raman measurements of intracellular ice, membrane partitioning, cellular material integrity or cytochrome C release. The −4°C ice nucleation and −3°C/min cooling were shown as the best combination of controlled rate freezing parameters for D7 SN. Post-thaw functional assay of cryopreserved D7 SN demonstrated a high-confluence, viable post-thaw culture (Figure 7C) that successfully produced the more mature neurons with normal electrophysiological responses to the panel of drug molecules (Figure 7D and Supplementary Figure S3).

**FIGURE 8:**
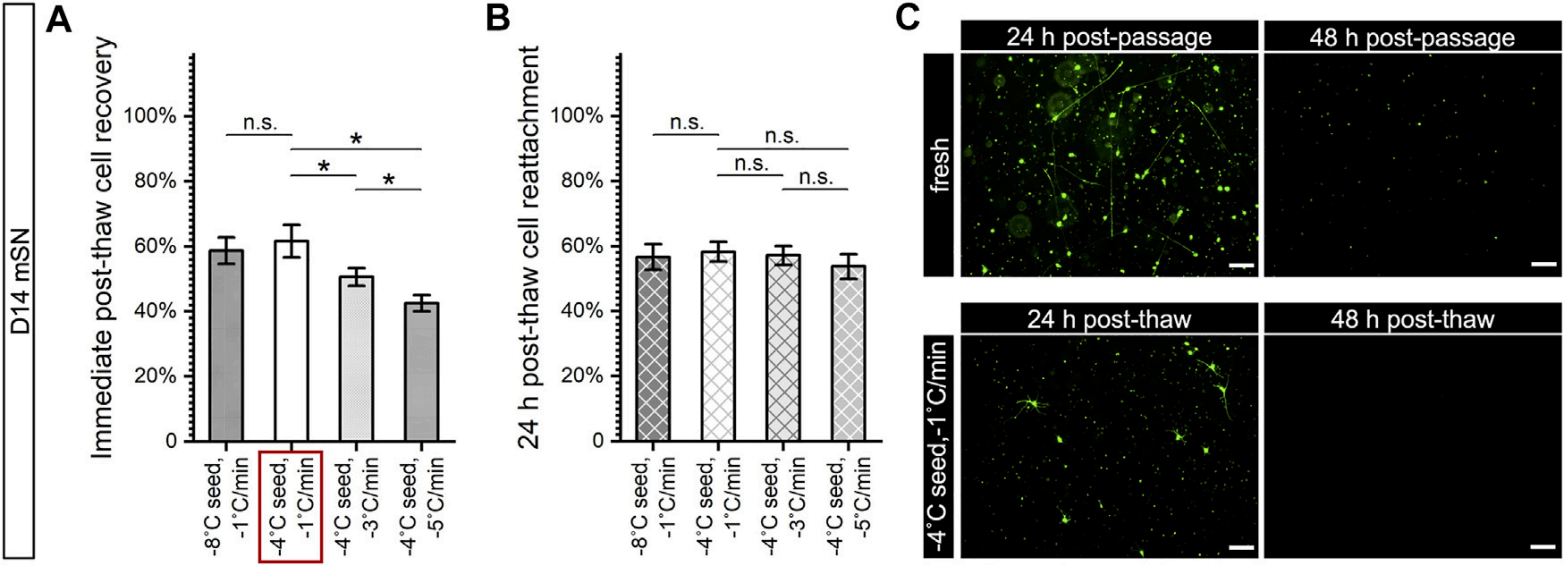
Post-thaw survival and function of cryopreserved D14 mSN under varying nucleation temperatures and cooling rates. Data are represented as mean ± 95% confidence interval. *p < 0.05; n.s.: *p* > 0.05. **(A)** Post-thaw recovery of D14 mSN showing no significant change with greater undercooling but trending significantly lower with faster cooling rate. *n* = 4. Red box: best-case parameters tested with the highest recovery. **(B)** Post-thaw reattachment of D14 mSN, normalized to reattachment of dissociated fresh cell control, showing no significant difference between any of the test conditions. *n* = 4. Crossed-out columns denoting insensitive post-thaw assay due to poor outcome of control cells. **(C)** Calcein AM-stained culture 24 and 48 h after replating dissociated (fresh) or cryopreserved (best-case) D14 mSN, showing a vast majority of live (calcein AM positive) cell population nonadherent or with rounded morphology in the first 24 h that was more sparsely distributed in the post-thaw culture, as well as subsequent nearly complete cell loss in both conditions at 48 h. Scale bar: 100 µm.

For cryopreserved D14 mSN, measurement of immediate post-thaw recovery (Figure 8A) appeared to demonstrate a lack of sensitivity to undercooling that was otherwise shown in the earlier Raman results, but it showed a trend of more cell death with faster cooling rate that was consistent with the trend in intracellular ice, membrane impairment, volumetric loss, and cytochrome C release observed by Raman. The 24-h post-thaw reattachment of D14 mSN (Figure 8B) also appeared to show a lack of sensitivity to both undercooling and cooling rate variations. However, upon a closer examination of D14 mSN post-thaw using the optimal freezing parameters (i.e., −1°C/min cooling and −4°C ice nucleation) as well as postpassage (Figure 8C), we found that both freshly dissociated and cryopreserved cells failed to produce viable culture, rendering 24-h post-thaw reattachment an invalid metric, in this instance, for quantifying the effect of cooling rate or undercooling on the cryopreservation outcome of D14 mSN.

## Discussion

In contrast to the abundant methods of primary and stem cell-derived neuronal cell production in literature, a few reports can be found to describe the cryopreservation of these cells. One of these few studies reported the cryopreservation of primary mouse cortical and dopaminergic neurons in DMSO-containing formulations and subsequent phenotypic characterization of the cryopreserved cells at 2 and 4 weeks post-thaw (Pischedda et al., 2018), without survival data obtained in shorter terms post-thaw, which was not directly comparable with results in the present study. Another study reported the cryopreservation of primary mouse motor neurons in DMSO and post-thaw cell recovery rate of 68.8% (Parker et al., 2018). There were two accounts of cryopreserved dopaminergic progenitors and neurons derived from hiPSCs and human embryonic stem cells (hESCs), with the highest reported post-thaw recovery approaching 50% upon 24-h treatment in apoptosis inhibitors (Wakeman et al., 2017; Drummond et al., 2020). In comparison, the present study demonstrated DMSO-free cryopreservation of neuronal cells, with post-thaw survival of D5 crest measured at 91.6% immediately post-thaw, 88.2% 24 h post-thaw, and of D7 SN measured at 93.6% immediately post-thaw, 84.1% 24 h post-thaw, all of which were significantly higher than 50% (*p* < 0.05) even without the use of apoptosis inhibitor. The methodology and results of this investigation represent a significant addition to the scientific literature related to the cryopreservation of hiPSC-derived sensory neurons and their progenitors. Its outcome may be validated using additional hiPSC lines, alternative neuronal differentiations and other tissue lineages.

### Cell Loss Before Freezing and After Thawing Challenges *in Vitro* Neuronal Cell Production

A commonly recognized process bottleneck in the developing supply chain of hiPSC-derived cryopreserved cell therapies has been the temporal restriction on the fill-and-finish step immediately preceding the freezing of cells to minimize cell loss due to DMSO cytotoxicity (Yuan et al., 2014; de Abreu Costa et al., 2017; Pollock et al., 2017; Verheijen et al., 2019). As demonstrated in Figure 2, the non-DMSO CPA molecules used in this study (i.e., sucrose, glycerol, isoleucine, P188, HSA) provided a superior alternative to the widely used DMSO in preserving cell viability before freezing. This result supports the transition from DMSO to non-DMSO in order to enable scale-up of the fill-and-finish process in a manufacturing setting, while adhering to a high batch consistency. Compared with DMSO whose cytotoxicity arises from dissolution, epigenetic and genomic alteration, these non-DMSO CPAs, typically with larger molecular weight than DMSO, can cause pre-freeze cell loss predominantly due to osmotic stress upon CPA introduction (Best, 2015; Hornberger et al., 2021; Matsumura et al., 2021). Compared with D5 crest and D7 SN, which exhibited no significant cell loss over the course of prefreeze exposure to the non-DMSO CPA solution (*p* > 0.05), D14 mSN experienced minor (10%) yet significant (*p* < 0.05) decrease in viable cell count during the first 30 min of this process, suggesting that sensitivity to the osmotic stress varied by cell stage with the most mature cells being most sensitive. Prefreeze CPA exposure testing in this study of these neuronal cells in the non-DMSO solution may be expanded in a future study to include longer times of exposure and determine the time limit for a given quality control (QC) range of cell viability.

### Membrane Fluidity Informs Undercooling Sensitivity and Cryopreservation Design

Literature in the field of gamete cryobiology has explored and demonstrated the inverse correlation between membrane fluidity and desirable cryopreservation outcome such as motility of recovered spermatozoa (Giraud et al., 2000; Blesbois et al., 2005). In addition, Noutsi et al. (2016) showed the varying extent of fluctuation in membrane fluidity among a selection of cell lines over the course of 4-day static cultures and spontaneous differentiation. These previous studies have required highly specialized spectrofluorometer or two-photon fluorescent microscopy to quantify membrane fluidity for their measurement, both of which are limited by low throughput and adaptability.

We found that membrane fluidity trended lower as differentiation and maturation progressed. The decrease in membrane fluidity was consistent with the increase in undercooling sensitivity of the cells. This result supports the correlation between membrane fluidity and cell freezing damage previously postulated in literature (Giraud et al., 2000; Blesbois et al., 2005) and provides more in-depth evidence specifying the nature of freezing damages as intracellular ice and cell disintegration consequent of extensive undercooling. On one hand, cells like D5 crest with very high membrane fluidity may exhibit little to no sensitivity to changes in ice nucleation temperature. On the other hand, cells like D14 mSN with low membrane fluidity would require a tight control of ice nucleation temperature (e.g., −3 to −7°C) in order to ensure the same level of consistency in their post-thaw cell survival and function.

One may note that despite having relatively higher membrane fluidity than hiPSC aggregates and utilizing the same non-DMSO CPA formulation, D14 mSN responded significantly more poorly to undercooling (Figures 3 and 6) than hiPSC aggregates (Li et al., 2020). Membrane fluidity alone may not reflect undercooling sensitivity of a given cell type. Other factors may include intracellular and transmembrane diffusion rates of water and the cell-penetrating CPA molecules (i.e., glycerol) for the given cell sample, as well as membrane partitioning of glycerol and its effect on the molecular interaction between intracellular CPA and water. With greater undercooling, kinetic energy and molecular motion are lower, and upon ice nucleation in the extracellular space, more rigid plasma membrane may not allow water molecules to transport quickly enough from the intracellular space following the sudden increase in the transmembrane concentration differential to avoid intracellular ice formation. Low membrane permeability may decrease the probability of water leaving the intermolecular hydrogen bond network and increase the probability of intracellular ice formation. Dense membrane-cytoskeletal organization may slow down the diffusion of water and CPAs in the cytoplasm and reduce the probability of uniform cryoprotection inside the cell. Shift in the membrane property associated with differentiation or aggregation of hiPSCs may change the partitioning of glycerol as observed by Raman and potentially deviate the local molecular balance from its sweet spot that was seen (Li et al., 2020) to mitigate undercooling stresses for hiPSCs.

It is also important to note that the non-DMSO solution was found to eliminate chunky, lethal intracellular ice formation regardless of the cell stage, whereas the DMSO-based solution was observed to result in significant amounts of such ice crystals when subjected to the same degree of undercooling. As membrane fluidity may drop as cells differentiate and mature, it becomes increasingly important to transition from DMSO, or a single-CPA non-DMSO alternative formulation, to a non-DMSO CPA cocktail like the one used in this study, in order to counter the intrinsic cell vulnerability to undercooling with proper cryoprotection and consequently improve the consistency of cryopreservation outcome and flexibility of CRF in hiPSC-based cell manufacturing.

### Cell Size Measurement Informs Cooling Rate Sensitivity and Cooling Rate Selection

It has been hypothesized and demonstrated in cryobiological literature (Gao and Critser, 2000; Hubel, 2018) that the relationship between cooling rate and cryopreservation outcome for a given cell type should resemble an inverted “U,” and that the optimal cooling rate of different cell types correlate with their difference in cell size. Within the cooling rate range (−1 to −5°C/min) tested in this study, the optimal cooling rate for D5 crest lay near or below −1°C/min, −3°C/min for D7 SN around, and for D14 mSN also near or below -1°C/min but skewed to a likely slower optimal cooling rate than that of D5 crest. This relativity in optimal cooling rate was consistent with their different cell sizes, where the larger cells (i.e., D14 mSN > D5 crest) require a slower cooling rate (i.e., −1°C/min and potentially lower) to avoid lethal intracellular ice formation.

Interestingly, compared with the nearly 50% drop in post-thaw reattachment of larger cell (D5 crest) along with the increase in cooling rate from −1 to −3°C/min, the decrease in cooling rate from −3 to −1°C/min resulted in less cell loss (i.e., 5% drop in post-thaw reattachment) in the smaller cell (D7 SN). Consistent with what was previously proposed (Gao and Critser, 2000), cooling rate sensitivity is most critical toward large cells with higher than optimal cooling rates. Insulative passive freezing devices and liquid nitrogen-free controlled rate freezers are limited by the typical maximum momentary cooling rate of −2°C/min, where freezing damage by fast cooling may not be a primary concern in their applications. In liquid nitrogen-based controlled rate freezing applications, measuring cell diameter in suspension by light microscopy may be useful to determine whether the cell form of interest requires a slower cooling rate to decrease the probability of freezing damages or relaxes the acceptable range to accommodate deviations toward faster cooling rates or intentional use of accelerated programs.

### Dissociation of Adherent Culture Remains a Challenge to Cell Cryopreservation

This investigation revealed a critical challenge to the successful cryopreservation of hiPSC-derived cells. The interruption of culture on day 5 created a barrier to neuronal induction of neural crest cells, and the 90-min Accumax treatment on day 14 failed to produce a viable culture of mature sensory neurons, with or without cryopreservation. Transcriptomic analyses in recent publications (Khan et al., 2016; Adam et al., 2017; Mattei et al., 2020; Miyawaki-Kuwakado et al., 2021) showed that enzymatic dissociation can alter the gene expression of cells in a fashion that is independent of the treatment concentration, duration, and temperature but dependent on the enzyme species. Therefore, in order to successfully cryopreserve intermediate cell stages of an hiPSC differentiation like D5 crest or advanced cell stages of maturation like D14 mSN, it will be critical to optimize the cell dissociation protocol, especially the molecular species if using an enzymatic method, minimizing cell loss, changes in the gene expression, and deviation from the intended cell developmental trajectory.

A recent study demonstrated vitrification of adherent hiPSC-derived neural progenitor cells using the TWIST method with 80% post-thaw recovery. While the methodology of vitrification is limited in scalability, advances as such in improving its sterility and future studies in validating the method for more differentiated, mature cell stages may present practical value toward *in situ* cryopreservation of hiPSC-derived 2D adherent culture at laboratory scale. Furthermore, while slow freezing has been predominantly used for cryopreserving single-cell suspensions, its feasibility for *in situ* cryopreservation of adherent cell sheet was recently demonstrated for readily implantable hiPSC-derived retinal pigment epithelium (Pennington et al., 2021). The 3D spheroid suspension culture of hiPSCs and differentiated cells has recently gained popularity due to its scalability demanded by preclinical and clinical cell production (Chen et al., 2015; Kwok et al., 2018; Takahashi et al., 2018). Cryopreservation of hiPSC-derived cell spheroids may be studied in the future to examine how the effect of cryopreservation on continuity of differentiation varies between different modalities.

## Data Availability

The original contributions presented in the study are included in the article/Supplementary Material. Further inquiries can be directed to the corresponding author.
