# Supplementary material for: Differentiation of Human iPS Cells Into Sensory Neurons Exhibits Developmental Stage-Specific Cryopreservation Challenges
Source: Front Cell Dev Biol. 2021 Dec 14;9:796960. doi: 10.3389/fcell.2021.796960 (PMC8712858; doi:10.3389/fcell.2021.796960)
Supplement: Supplementary file 1 [file DataSheet1.docx]

Supplementary Material

# Supplementary Table and Figures

**Supplementary Table 1.** Raman spectral peak assignments^a^

| **Substance** | **Wavenumber (cm^-1^)** | **Assignments** (Selvarajan, 1966; Adar et al., 1978; Mathlouthi and Vinh Luu, 1980; Mendelovici et al., 2000; Stone et al., 2004; Salzmann et al., 2006; Okotrub and Surovtsev, 2013; Zhan et al., 2021) |
| --- | --- | --- |
| Cell | 77 - 154 | Hydrogen bond vibration |
| Protein | 1622 - 1699 | Amide I |
| Cytochrome C | 1117 - 1137 | Heme vibration |
| Ice | 195 - 235 | Hydrogen bond vibration |
| Ice | 3087 - 3162 | OH stretching |
| Non-DMSO CPAs (i.e., sucrose, glycerol, isoleucine, P188, HSA)* | 815 - 865 | CC stretching |
| DMSO | 648 - 726 | Symmetric CS stretching |

*^a^DMSO, dimethyl sulfoxide; CPA, cryoprotective agent; P188, poloxamer 188; HSA, human serum albumin; OH, CC, CS refer to chemical bonds.*

**abbreviated and labeled in Raman images as “CPA”*

**
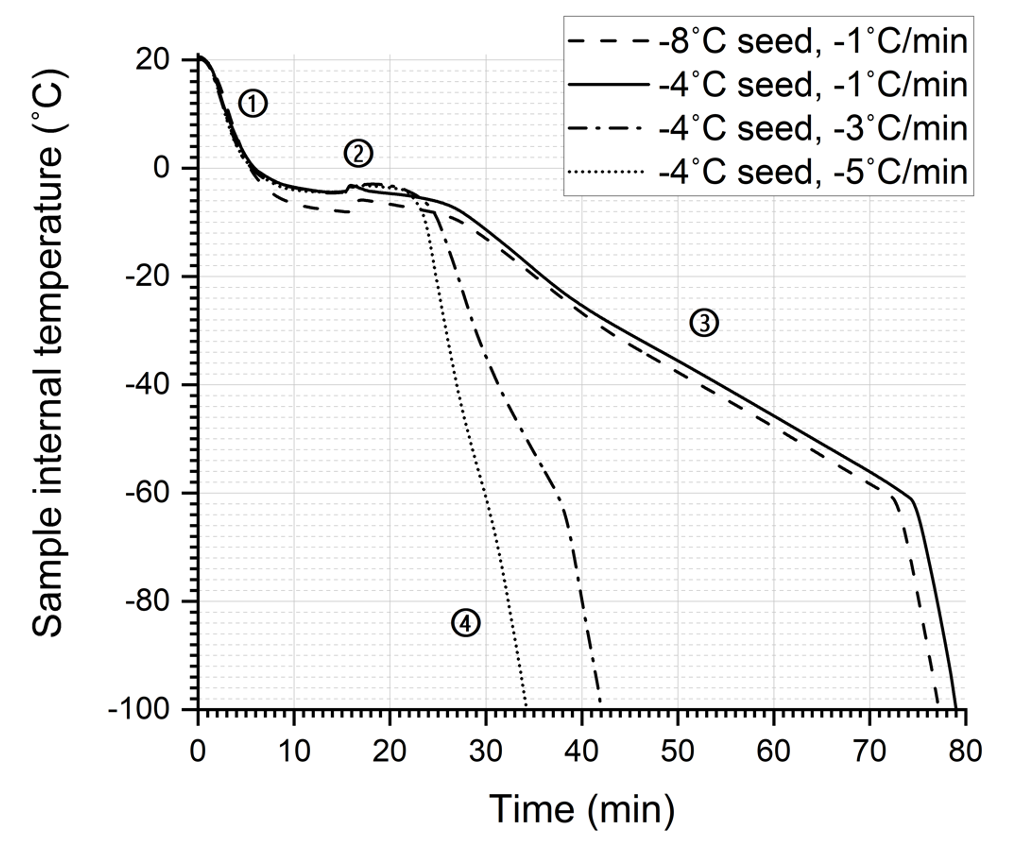
**

**Supplementary Figure 1.** Sample temperature profiles during controlled rate freezing varied by three different cooling rates and two different ice nucleation temperatures. Step 1 represented gradual cooling to the intended ice nucleation temperature. Step 2 represented a subtle increase in sample temperature as the result of latent heat of fusion upon induced ice nucleation (“seed”). Step 3 represented slow cooling at the intended cooling rate and ice growth phase. Step 4 represented fast cooling to the final sample temperature of -100˚C before samples were transferred to cryogenic storage.


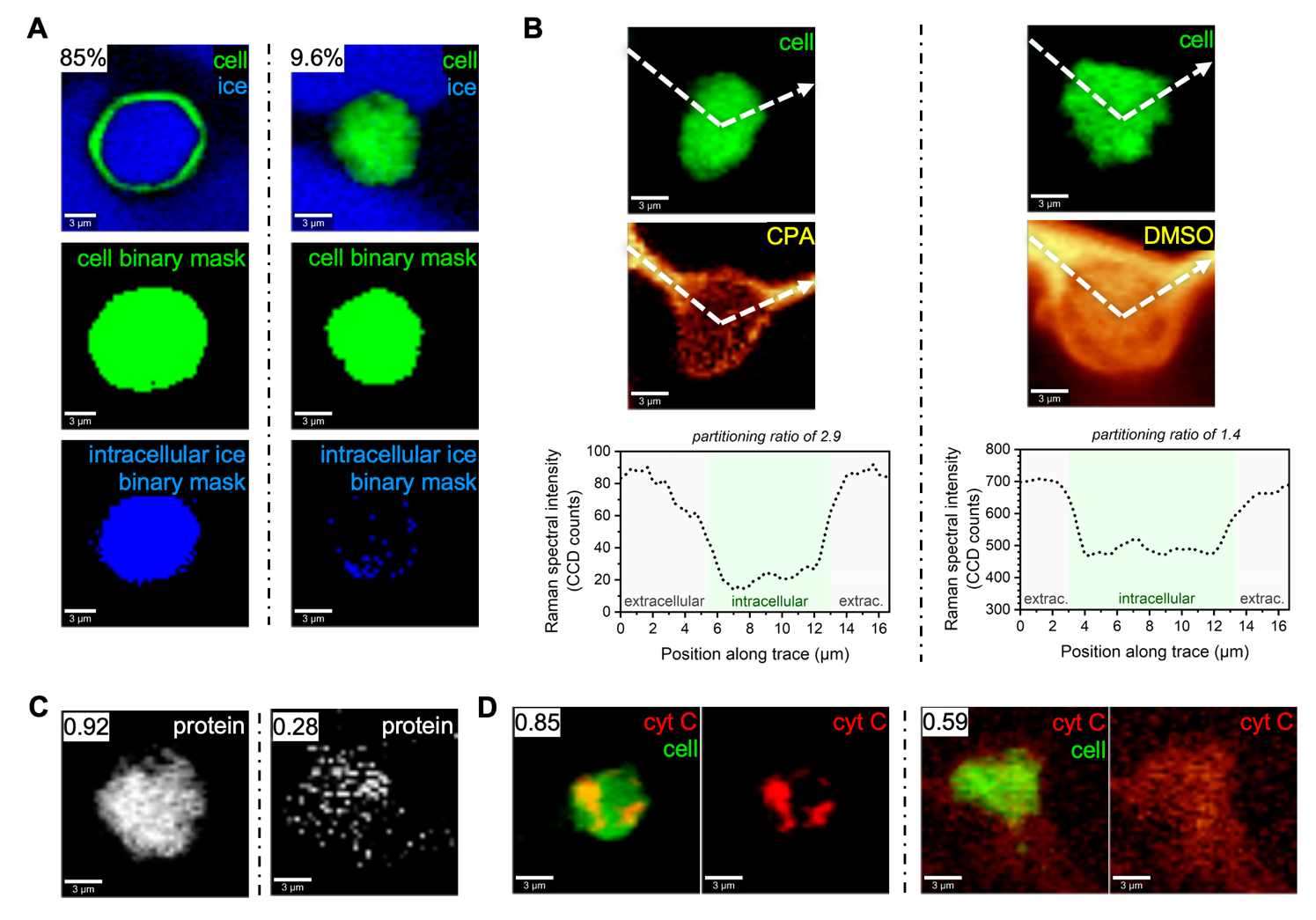


**Supplementary Figure 2**. Illustrative examples of quantitative Raman metrics used in this investigation. Scale bar: 3 µm. (**A**) Contrast of two samples with high (left) versus low (right) intracellular ice formation. Overlay and binary mask of Raman heat maps of cell and ice with area proportion of ice inside cell (AIC) values displayed per sample. (**B**) Contrast of two samples with high (left) versus low (right) membrane partitioning of non-DMSO CPA molecules versus DMSO. Raman heat maps and CPA (or DMSO) concentration profile traced along the dotted segmented arrow with partitioning ratio values displayed per sample. (**C**) Contrast of two samples with normal versus disintegrated cellular proteins. Raman heat map of protein by amide I signal with spatial autocorrelation value displayed per sample. (**D**) Contrast of two samples with cytochrome C (cyt C) contained in (left) versus released from (right) mitochondrial region. Overlay of Raman heat maps of cyt C and cell, as well as a heat map of cyt C alone that was used for Moran’s I analysis, with spatial autocorrelation value of cyt C displayed per sample.

**
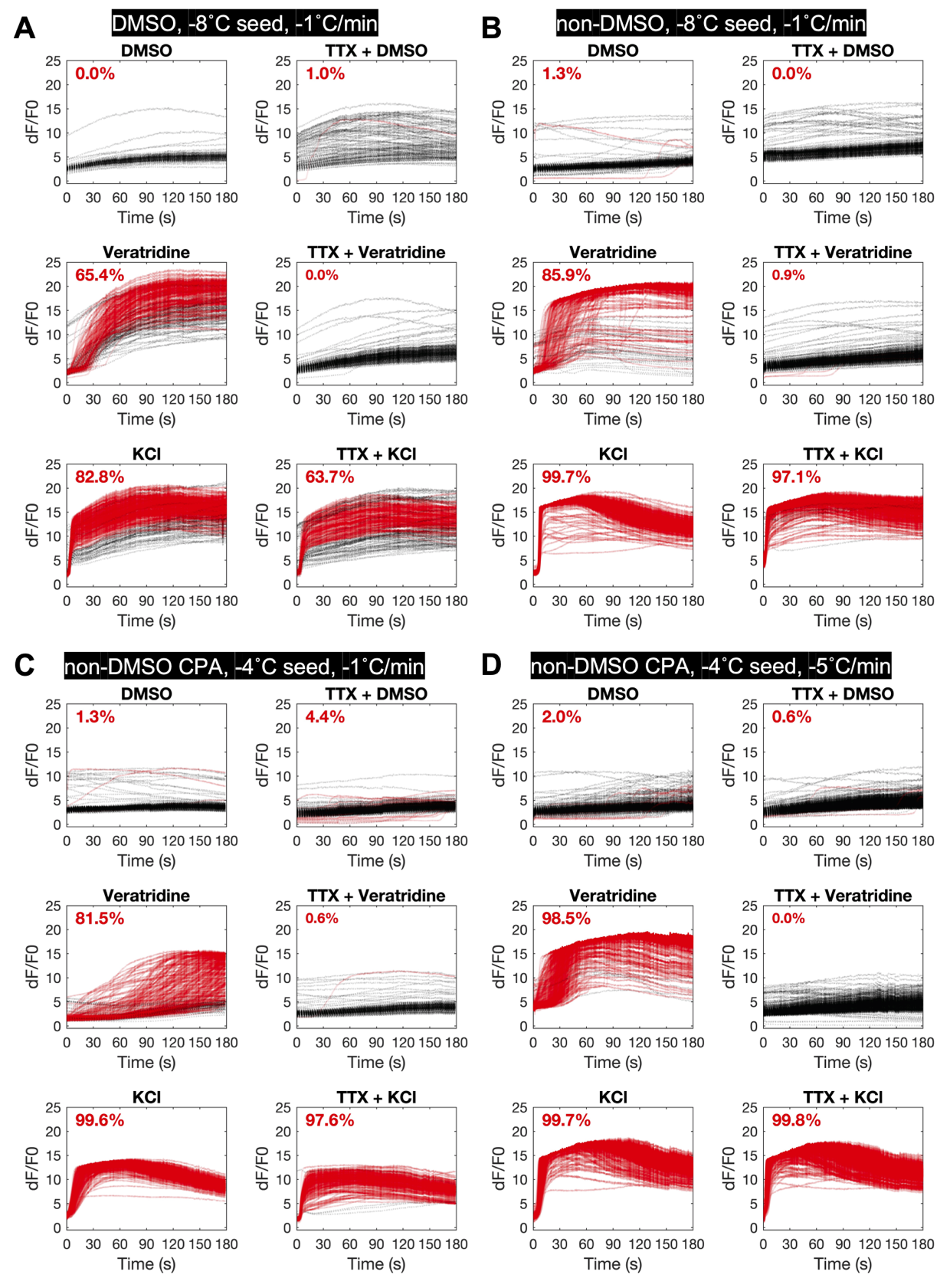
**

**Supplementary Figure 3**. Calcium imaging of post-thaw culture after 7-day maturation of D7 SN cryopreserved in DMSO versus non-DMSO solutions, under varying nucleation temperatures and cooling rates, in addition to Figures 1D and 6D. All non-DMSO conditions showed little to no response to 0.1% DMSO (negative control), positive response to 1 µM veratridine that was inhibited by TTX, and positive response to 30 mM KCl that was largely unaffected by TTX, whereas the DMSO condition showed fractionally lower proportion responding to KCl that was also fractionally reduced upon TTX treatment. Red line: responder cell; black line: non-responder cell. Proportion of responder cell population indicated per graph. Range of *n* = 204 – 544.
